# Prevalence and Correlates of Self‐Stigma in Personality Disorder Compared With Anxiety and Depression: A National Cross‐Sectional Survey

**DOI:** 10.1002/pmh.70011

**Published:** 2025-03-13

**Authors:** Ruksana Begum‐Meades, Sophie Feilder, Mike J. Crawford

**Affiliations:** ^1^ Division of Psychiatry Imperial College London London UK

**Keywords:** internalised stigma, mental health, personality, personality disorder, self‐stigma

## Abstract

Self‐stigma occurs when a person with a mental illness internalises the negative stereotypes and attitudes associated with their condition, which can lead to reduced help‐seeking and social withdrawal. Previous research has demonstrated high levels of professional stigma towards people with personality disorder, but in contrast to mental disorders such as anxiety and depression, very little is known about self‐stigma in people with personality disorder. We conducted an online, cross‐sectional survey of 1009 people who had received a diagnosis of personality disorder, anxiety or depression to compare levels of self‐stigma and identify associated factors. We assessed self‐stigma using the Internalised Stigma of Mental Illness Scale‐9 and demographic and clinical factors including level of personality disturbance, invalidating childhood experiences and depressive symptoms. In multilevel analysis, people diagnosed with personality disorder did not have higher levels of self‐stigma than those with anxiety and depression. Levels of self‐stigma were higher among those with higher levels of personality disturbance, depression and invalidating childhood experiences. These findings highlight the importance of personality disturbance in the development of self‐stigma and the need for interventions to increase mental health literacy in this area.

## 1 Introduction

Stigma can be conceptualised as prejudicial views held about a group of people, with society viewing individuals as part of this group in a negative light (Corrigan and Rao 2012; Goffman 1963). People in a stigmatised group may internalise these views: This phenomenon is known as self‐stigma (Corrigan and Rao 2012).

Research investigating self‐stigma in people with mental health problems has primarily focused on people with severe mental illnesses such as schizophrenia and psychosis (Quinn, Williams, and Weisz 2015) and common mental conditions such as depression (Barney et al. 2010; Grant, Bruce, and Batterham 2016; Kanter, Rusch, and Brondino 2008). This research has found that factors such as severity of depressive symptoms, duration of mental ill health and level of education are associated with self‐stigma (Gilkes, Perich, and Meade 2019; Grambal et al. 2016; Holubova et al. 2021; Quenneville et al. 2020; Quinn, Williams, and Weisz 2015). Childhood trauma also appears to be associated with greater levels of self‐stigma with research having looked at people with alcohol use disorders, in part due to the greater likelihood of agreeing with negative stereotypes that may occur as a result of these early experiences (Stolzenburg et al. 2018).

Self‐stigma is associated with a range of negative consequences including but not limited to reduced self‐esteem, engaging in avoidance behaviours and feeling unable to work towards life goals (Corrigan and Rao 2012; Wright, Haigh, and McKeown 2007). Higher levels of self‐stigma may also contribute to pessimistic attitudes towards treatment (Corrigan 2004; Lannin et al. 2016; Vogel, Wade, and Hackler 2007). This may in turn lead to withdrawal from therapeutic interventions, increasing the risk to an individual's well‐being if they do not receive the support or treatment they may need (Ociskova et al. 2017). Greater understanding of the origins of self‐stigma is needed, if appropriate interventions are to be developed to reduce self‐stigma and its negative consequences.

Stigma related to personality disorder is pervasive and has wide‐ranging impacts, from access to services and appropriate psychological support (Dale et al. 2017), experiences interacting with healthcare professionals (Carrotte, Hartup, and Blanchard 2019) and overall well‐being (Chan et al. 2022; Corrigan, Larson, and Rüsch 2009). The shift to the new ICD‐11 system for diagnosing and classifying personality disorder represents a significant change in the field of mental health. This updated approach moves away from the traditional categorical model as seen in the DSM‐5 and earlier editions of the ICD to a dimensional framework that offers a more nuanced understanding of personality disorder. As such, the ICD‐11 now conceptualises personality disorder along a continuum, ranging from mild to severe personality disorder. This new structure allows for a more accurate representation of the spectrum of personality disorder that is frequently observed in clinical practice, providing a more flexible and accurate tool to diagnose and treat individuals.

Most research on stigma in personality disorder has focused on professional stigma, with previous research repeatedly highlighting negative attitudes held by many clinicians, such as viewing people with personality disorder as manipulative, in control of their emotions, difficult to treat and viewing personality disorder itself as a behavioural issue, not a mental health problem (Baker and Beazley 2022; Lewis and Appleby 1988; Newton‐Howes, Weaver, and Tyrer 2008). Concerns have also been expressed that professional stigma may influence the way that people diagnosed with personality disorder may feel about themselves and the impact that this may have on the treatment and support they receive (Aviram, Brodsky, and Stanley 2006; Klein, Fairweather, and Lawn 2022).

Although there is a large body of research on professional stigma towards people with personality disorder, self‐stigma among people with personality disorder has received far less attention to date. The existing literature on self‐stigma in people with personality disorder has begun to provide some valuable insights. Studies have looked to explore the prevalence and correlates of self‐stigma in people with personality disorder. Research by Grambal et al. (2016) showed that individuals with a diagnosis of borderline personality disorder experience higher levels of self‐stigma compared with those with a diagnosis of other mental health conditions, such as schizophrenia, bipolar disorder, depression and anxiety. Self‐stigma has also been associated with negative outcomes in this population, including reduced self‐esteem, increased symptom severity and a reduction in help‐seeking behaviours (Ociskova et al. 2023; Rüsch et al. 2006). Furthermore, Catthoor et al. (2015) examined stigma in adolescents with personality disorder. The authors reported that stigma is greater in adolescents with personality disorder compared with those without the diagnosis, with this increasing further as severity of personality disorder increases. Catthoor et al. posit that adolescents may be more vulnerable to internalising stigmatising attitudes, serving as a starting point in understanding personality disorder‐related stigma among younger age groups.

Despite these valuable contributions, the current body of research on self‐stigma in people with personality disorder comes with its limitations. Most studies have thus far focused solely on borderline personality disorder. Studies have generally relied on small sample sizes, which may limit the generalisability of the findings obtained. Finally, contextual factors, such as experiences of navigating healthcare systems, should also be considered because of the potential impact of professional stigma on the development or severity of self‐stigma. Understanding these areas further is crucial for developing future strategies to address self‐stigma at both the individual and societal levels.

We therefore set out to investigate self‐stigma in people with and without a self‐reported diagnosis of personality disorder who have had contact with mental health services.

The objectives of the study were to

- Compare levels of self‐stigma among people with personality disorder with those of people with depression and anxiety.
- Examine factors, including the level of personality difficulty, invalidating childhood experiences and experiences of contact with mental health services, that may influence levels of self‐stigma among people with personality disorder.
- Explore the relationship between self‐stigma and attitudes towards treatment among people with personality disorder.

## 2 Method

### 2.1 Study Participants

We conducted a national cross‐sectional online survey of people in the United Kingdom who reported having had contact with NHS mental health services and being diagnosed with personality disorder, depression or anxiety. To take part in the study, potential participants needed to be aged 18 or over, have had contact with NHS mental health services and have been given a diagnosis of personality disorder, depression or anxiety. People living outside of the United Kingdom, those who had a previous diagnosis of psychosis and those who were unable or unwilling to provide informed consent were excluded from the survey. Individuals who had not had prior contact with NHS mental health services were also excluded. This is because we wanted to examine the impact that contact with mental health services, including being diagnosed with personality disorder, may have on levels of self‐stigma.

### 2.2 Study Recruitment

Study participants were recruited via social media, with adverts placed on Facebook, Instagram and Facebook Messenger. We ran the survey online using Qualtrics software (www.qualtrics.com). Potential participants who were interested in taking part in the study were asked to read an online copy of the Participant Information Sheet, before deciding whether to provide electronic consent and completing the survey. People who did not provide informed consent, were located outside of the United Kingdom (determined by Qualtrics' GeoIP location filter), reported a diagnosis of psychosis or did not have any previous contact with NHS mental health services were excluded from the study.

### 2.3 Measures Used

The primary outcome measure was the *Internalised Stigma of Mental Illness Scale‐9* (ISMI‐9) (Hammer and Toland 2017). People completing the scale are asked to rate the extent to which they agree or disagree with five concepts associated with self‐stigma: alienation, social withdrawal, stereotype endorsement, perceived discrimination and stigma resistance (Hammer and Toland 2017; Ritsher, Otilingam, and Grajales 2003). Scores are calculated by adding together the item scores and dividing by the number of answered items. Ritsher and Phelan's (2004) two‐category method for interpreting ISMI‐9 results states that scores above 2.51 indicate high self‐stigma. Internal consistency of the ISMI‐9 was found to be excellent, with a Cronbach's alpha of 0.86 (Hammer and Toland 2017).

We assessed the level of personality pathology using the *Standard Assessment of Personality‐Abbreviated Scale* (SAPAS) (Moran et al. 2003). The SAPAS consists of nine items taken from the Standard Assessment of Personality (Mann et al. 1981). Items are answered either ‘yes’ or ‘no’ and assigned a score of 0 or 1. A score of 4 or more indicates that personality disorder may be present in general community samples (Fok et al. 2015). Internal consistency of the SAPAS was considered to be acceptable, with a Cronbach's alpha of 0.68 (Moran et al. 2003). We used the *Patient Health Questionnaire‐2* (PHQ‐2) (Kroenke, Spitzer, and Williams 2003) to assess the presence and severity of depressive symptoms. Although internal consistency was not reported for the PHQ‐2, a score of ≥ 3 was found to have a sensitivity of 83% and a specificity of 92%. Furthermore, the PHQ‐9, on which this measure is based, has excellent internal consistency, with a Cronbach's alpha of 0.89 (Kroenke, Spitzer, and Williams 2001). We used the *Invalidating Childhood Environment Scale‐9* (ICES‐9) (Robertson, Kimbrel, and Nelson‐Gray 2013) to retrospectively assess childhood parental invalidation. Each item is rated from ‘never’ to ‘all of the time’ and scored from 1 to 5. Total invalidation scores are calculated by adding the item scores together, with higher scores indicating greater exposure to invalidating childhood experiences. Although the internal consistency of the ICES‐9 has not been reported, the original ICES measure reports excellent internal consistency (maternal invalidation *α* = 0.90, paternal invalidation *α* = 0.88; Robertson, Kimbrel, and Nelson‐Gray 2013). Furthermore, the ICES‐9 has been shown to have improved factor structure compared with the original ICES (Robertson, Kimbrel, and Nelson‐Gray 2013), as well as being shorter and therefore quicker to administer.

Study participants were asked to provide demographic information including age, gender, ethnicity, level of education and occupational status. They were also asked about contact with healthcare services including their mental health diagnosis, the number of appointments and years of contact with NHS mental health services and the type of services they had used (e.g., primary care or secondary care services).

### 2.4 Sample Size

The sample size calculation was based on the primary hypothesis: that self‐stigma would be greater in people with a personality disorder diagnosis, compared with those with a diagnosis of depression or anxiety. Based on the findings of Boyd, Otilingam, and DeForge (2014) that approximately 30% of individuals with a diagnosed mental health condition experience moderate to high levels of self‐stigma, a sample size of 920 was needed, to detect an odds ratio of 1.6, with 90% power and 0.05 level of statistical significance.

### 2.5 Data Analysis

Data were analysed on IBM SPSS Statistics, Version 29. We used appropriate univariate statistics to assess the association between exposure variables and the level of self‐stigma. Having checked that the requirements and assumptions for conducting regression analysis were met, we then used linear regression analysis to generate a model of clinical and demographic factors that best explained variation in the level of self‐stigma. To avoid overfitting the model, the factors that were selected for inclusion for multivariate analysis were chosen based on previous research, as well as the results of the preliminary univariate analysis. For the main analysis, we created a binary categorical variable of whether a participant reported having been given a diagnosis of personality disorder. When a participant stated that they had been diagnosed with both personality disorder and other common mental disorders, we categorised them as having had a diagnosis of personality disorder.

### 2.6 Ethical Approval

Ethical approval was granted by Imperial College Research Ethics Committee (ICREC reference number: 6402211, 13 March 2023) prior to the start of data collection.

## 3 Results

Between 15 March and 4 April 2023, 1497 people completed an eligibility assessment. Of these, 1009 were eligible and completed the survey and 469 (31.7%) were excluded—mainly because they had been diagnosed with psychosis (*n* = 283, 18.9%) or did not provide informed consent (*n* = 93, 6.2%).

### 3.1 Demographic Characteristics

Most people who took part in the study were female (*n* = 874, 86.6%), 76 (7.5%) were male, and 59 (5.9%) indicated other. The average age of participants was 38.06 (SD = 13.33). Level of education varied, with 35.9% of participants at further education level and 32.2% having obtained an undergraduate degree. The majority of participants were White British (*n* = 925, 91.7%), 14 (1.4%) were Asian British, and 5 (0.5%) were Black British. Among the study sample, 378 (37.5%) reported having been given a diagnosis of personality disorder, and 631 (62.5%) had not. Demographic and clinical characteristics of people given a diagnosis of personality disorder, compared with those who had not, are presented in Table 1 below.

**TABLE 1:** Table of participant characteristics.

Characteristic	Category	PD diagnosis (*n* = 378)	No PD diagnosis (*n* = 631)	Total (*n* = 1009)
Gender	Male	22 (5.8%)	54 (8.6%)	76 (7.5%)
	Female	328 (86.8%)	546 (86.5%)	874 (86.6%)
	Other	28 (7.4%)	31 (4.9%)	59 (5.9%)
Age (SD)		35.34 (11.80)	39.69 (13.93)	38.06 (13.33)
Ethnicity	White British	338 (89.4%)	587 (93.0%)	925 (91.7%)
	Asian British	5 (1.3%)	9 (1.4%)	14 (1.4%)
	Black British	4 (1.1%)	1 (0.2%)	5 (0.5%)
	Mixed or multiple ethnic groups	9 (2.4%)	5 (0.8%)	14 (1.4%)
	White other	22 (5.8%)	28 (4.4%)	50 (4.9%)
	Other		1 (0.2%)	1 (0.1%)
Level of education	Primary education	8 (2.1%)	5 (0.8%)	13 (1.3%)
	Secondary education	53 (14%)	112 (17.7%)	165 (16.4%)
	Further education	156 (41.3%)	206 (32.6%)	362 (35.9%)
	Undergraduate degree	121 (32%)	204 (32.3%)	325 (32.2%)
	Master's degree	29 (7.7%)	88 (13.9%)	117 (11.6%)
	Doctorate	5 (1.3%)	9 (1.4%)	14 (1.4%)
	No response	6 (1.6%)	7 (1.1%)	13 (1.3%)
Occupational status	Part time	58 (15.3%)	93 (14.7%)	151 (15.0%)
	Full time	89 (23.5%)	219 (34.7%)	308 (30.5%)
	Unemployed	179 (47.4%)	192 (30.4%)	371 (36.8%)
	Student	38 (10.1%)	66 (10.5%)	104 (10.3%)
	Retired	14 (3.7%)	61 (9.7%)	75 (7.4%)
Additional diagnoses	cPTSD	132 (34.9%)	141 (22.3%)	273 (27.1%)
	ASD	55 (14.6%)	81 (12.8%)	136 (13.5%)
	ADHD	28 (7.4%)	40 (6.3%)	68 (6.7%)
	OCD	34 (9%)	60 (9.5%)	94 (9.3%)
Advised to seek support for problem drinking/ substance misuse		90 (23.8%)	61 (9.7%)	151 (15%)
Number of appointments with mental health services	1 appointment	7 (1.9%)	20 (3.2%)	27 (2.7%)
	2–5 appointments	14 (3.7%)	92 (14.6%)	106 (10.5%)
	6–10 appointments	23 (6.1%)	108 (17.1%)	131 (13.0%)
	11–20 appointments	24 (6.3%)	92 (14.6%)	116 (11.5%)
	More than 20 appointments	310 (82.0%)	319 (50.6%)	629 (62.3%)
Years of contact with mental health services	Less than 1 year	12 (3.2%)	86 (13.6%)	98 (9.7%)
	1–2 years	34 (9.0%)	100 (15.8%)	134 (13.3%)
	3–5 years	62 (16.4%)	126 (20.0%)	188 (18.6%)
	More than 5 years	270 (71.4%)	319 (50.6%)	589 (58.4%)
Primary or secondary care	Only with talking therapies (IAPT) services	16 (4.2%)	202 (32.0%)	218 (21.6%)
	Only with staff working in CMHTs	82 (21.7%)	88 (13.9%)	170 (16.8%)
	A mix of contacts with these types of services	280 (74.1%)	341 (54.0%)	621 (61.5%)
Contact with lived experience practitioners		124 (38.9%)	115 (18.2%)	239 (23.7%)
Inpatient admission	No	183 (48.4%)	546 (86.5%)	729 (72.2%)
	1 admission	55 (14.6%)	55 (8.7%)	110 (10.9%)
	2 admissions	33 (8.7%)	14 (2.2%)	47 (4.7%)
	3 or more admissions	107 (28.3%)	16 (2.5%)	123 (12.2%)
PHQ‐2 score	MDD likely (> 3)			
SI‐MH score (SD)		28.78 (11.18)	34.79 (12.06)	32.53 (12.09)
ICES‐9 score (SD)		27.85 (8.37)	24.55 (8.28)	25.79 (8.46)
ISMI‐9 score	High internalised stigma (> 2.51)	271 (71.7%)	360 (57.1%)	631 (62.5%)
SAPAS score	Mild (> 4)	330 (87.3%)	485 (76.9%)	815 (80.8%)
	Moderate (> 5)			

*Note:* Data have been displayed either as *n* (percentage of participants) for categorical variables or mean (standard deviation) for continuous variables.

Abbreviations that have been used within the table are as follows: ADHD (attention deficit hyperactivity disorder); ASD (autism spectrum disorder); CMHT (Community Mental Health Teams); cPTSD (complex post‐traumatic stress disorder); IAPT (Improving Access to Psychological Therapies); ICES‐9 (Invalidating Childhood Experiences Scale‐9); ISMI (Internalised Stigma of Mental Illness‐9); MDD (major depressive disorder); OCD (obsessive‐compulsive disorder); PHQ‐2 (Patient Health Questionnaire‐2); SAPAS (Standard Assessment of Personality‐Abbreviated Scale); SI‐MH (Satisfaction Index‐Mental Health).

### 3.2 Level of Self‐Stigma

Overall, the mean score on the ISMI‐9 was 2.68 (SD = 0.55). In total 631 (62.5%) had a score of ≥ 2.51, indicating a high level of self‐stigma. Mean ISMI‐9 in people who had been given a diagnosis of personality disorder was 2.81 (0.53), compared with 2.60 (SD = 0.55) among those who had not (difference in means = 0.21, 95% CI = [0.14, 0.28], *p* < 0.001).

### 3.3 Univariate Analysis of Factors Associated With Self‐Stigma

Univariate analysis was conducted to identify factors associated with levels of self‐stigma. The reference category for each of the categorical variables was that which contained the largest number of participants per variable. Factors associated with levels of self‐stigma were age, with a weak negative correlation (*r* = −0.099, *p* = 0.002). Level of self‐stigma was significantly higher in those with only primary level education, compared with those who had reached up to further education (*t*(373) = −2.886, *p* = 0.002), but significantly lower in those with a higher education degree (Undergraduate degree, *t*(685) = 5.696, *p* < 0.001; Master's degree, *t*(477) = 5.569, *p* < 0.001; Doctorate, *t*(374) = 2.450, *p* = 0.007). Compared with being unemployed, being in employment or being a student was found to be associated with significantly lower levels of self‐stigma (Part time, *t*(520) = 7.014, *p* < 0.001; Full time, *t*(677) = 11.079, *p* < 0.001; Student, *t*(473) = 3.668, *p* < 0.001), as well as being retired (*t*(444) = 6.995, *p* < 0.001).

The level of self‐stigma was found to be significantly higher in people with a diagnosis of personality disorder, compared with those without, *t*(1007) = 5.885, *p* < 0.001. There was a small but significant positive correlation between the ICES‐9 score and self‐stigma (*r* = 0.252, *p* < 0.001). There was a moderate positive correlation between PHQ‐2 score and levels of self‐stigma (*r* = 0.506, *p* < 0.001), indicating that the level of self‐stigma increased as depression severity increased. A significant difference in levels of self‐stigma was found when comparing the means of participants with a SAPAS score of ≥ 4, compared with those with a SAPAS score of < 4 (*t*(1007) = 14.098, *p* < 0.001), with participants with a SAPAS score of ≥ 4 demonstrating higher levels of self‐stigma.

Compared with having more than 20 appointments with NHS mental health services, self‐stigma was found to be significantly lower in participants who had only had one appointment (*t*(654) = 2.524, *p* = 0.006), had two to five appointments (*t*(733) = 2.721, *p* = 0.003), had six to 10 appointments (*t*(758) = 3.563, *p* < 0.001) or had 11–20 appointments (*t*(743) = 1.754, *p* = 0.040). Having had less than 1 year of contact with NHS mental health services was associated with significantly lower levels of self‐stigma compared with having more than 5 years of contact, *t*(685) = 2.209, *p* = 0.014. In the same vein, having 1–2 years (*t*(721) = 2.010, *p* = 0.022) or 3–5 years (*t*(775) = 1.820, *p* = 0.035) of contact with mental health services was associated with significantly lower levels of self‐stigma. Only having contact with IAPT services was associated with lower levels of self‐stigma compared with having contact with a mix of service types, *t*(837) = 4.279, *p* < 0.001. Compared with not having psychiatric inpatient admission, being admitted to hospital once (*t*(837) = −2.063, *p* = 0.020) or three or more times (*t*(850) = −2.226, *p* = 0.013) was associated with higher levels of self‐stigma.

### 3.4 Multivariate Analysis of Factors Associated With Self‐Stigma

The results of the multivariate analysis are presented in Table 2. The model examined the main effects of a combination of factors on levels of self‐stigma, and a significant model emerged, *F*(20, 1008) = 36.678, *p* < 0.001, explaining 42.6% of the variance in levels of self‐stigma.

**TABLE 2:** Multivariate analysis of factors associated with self‐stigma.

		B	Standard error	*β*	*p*‐value
(Constant)		19.395	0.847		
PD diagnosis		0.305	0.298	0.030	0.305
SAPAS	≥ 4	3.023	0.325	0.239	**< 0.001**
PHQ‐2 score		0.973	0.066	0.375	**< 0.001**
ICES‐9 score		0.047	0.015	0.079	**0.002**
Education	Primary education	2.442	1.083	0.055	**0.024**
	Undergraduate	−1.064	0.279	−0.100	**< 0.001**
	Master's	−1.256	0.405	−0.081	**0.002**
	Doctorate	−1.339	1.057	−0.031	0.205
Occupation	Part time	−1.467	0.381	−0.105	**< 0.001**
	Full time	−2.282	0.322	−0.211	**< 0.001**
	Student	−1.518	0.466	−0.093	**0.001**
	Retired	−1.715	0.553	−0.090	**0.002**
Inpatient admission	1 admission	−0.418	0.358	−0.038	0.244
	3+ admissions	−0.686	0.474	−0.045	0.148
Age		0.018	0.012	−0.048	0.121
Years of contact	Less than 1 year	0.400	0.453	0.024	0.377
Type of contact	IAPT only	−0.126	0.328	−0.010	0.701
Contact with services	1 appointment	−0.687	0.802	−0.022	0.392
	2–5 appointments	−0.654	0.435	−0.040	0.133
	6–10 appointments	−0.495	0.385	−0.033	0.199

*Note: R* ^2^ value = 0.426, adjusted *R* ^2^ value = 0.414. Reference category for PD diagnosis = no diagnosis; SAPAS = < 4; Education = further education; Occupation = unemployed; Inpatient admission = no admission; Years of contact = > 5 years; Type of contact = mix of contact; Contact with services = > 20 appointments.

In the multivariate model, personality disorder diagnosis was not a significant predictor of levels of self‐stigma. In this model, factors significantly associated with higher levels of self‐stigma were SAPAS score ≥ 4 (*β* = 0.239, *p* < 0.001), PHQ‐2 score (*β* = 0.375, *p* < 0.001), ICES‐9 score (*β* = 0.079, *p* = 0.002) and level of education including primary education (*β* = 0.055, *p* = 0.024). Factors significantly associated with lower levels of self‐stigma were level of education including an undergraduate degree (*β* = −0.100, *p* < 0.001), a Master's degree (*β* = −0.081, *p* = 0.002), occupational status including working part time (*β* = −0.105, *p* < 0.001), full time (*β* = −0.211, *p* < 0.001), being a student (*β* = −0.093, *p* = 0.001) and retired (*β* = −0.090, *p* = 0.002). Although people who had been given a diagnosis of personality disorder had higher SAPAS scores (5.48 compared with 4.86, difference in means = 0.62, 95% CI = [0.41, 0.84]), the two variables were not collinear.

### 3.5 The Association Between Levels of Self‐Stigma and Attitudes Towards Treatment

Finally, we examined the relationship between ISMI‐9 scores and attitudes to treatment. As seen in Table 3 below, positive attitudes towards treatment were associated with lower levels of self‐stigma, such as the belief that taking medication for mental health problems is beneficial (Attitude 1 response ‘strongly agree’, *t*(767) = 2.186, *p* = 0.015, reference category ‘agree’) and that talking treatments for people with mental health problems could also be beneficial (Attitude 2 response ‘strongly agree’, *t*(911) = 3.266, *p* < 0.001, reference category ‘agree’).

**TABLE 3:** Univariate analysis of the association between self‐stigma and attitudes towards treatment.

Variable	Category	Mean ISMIscore (SD)	Meandifference/Correlationcoefficient	Confidenceinterval	*p*‐value
Attitude 1: Taking medication for mental health problems is helpful.	Agree	2.68 (0.53)	—	—	—
	Strongly agree	2.59 (0.58)	0.09	[0.01, 0.17]	**0.015**
	Disagree	2.79 (0.51)	−0.11	[−0.21, −0.02]	**0.008**
	Strongly disagree	2.75 (0.67)	−0.07	[−0.21, 0.07]	0.161
Attitude 2: Talking treatments are beneficial for people with mental health problems.	Agree	2.69 (0.52)	—	—	—
	Strongly agree	2.57 (0.59)	0.12	[0.05, 0.19]	**< 0.001**
	Disagree	2.95 (0.49)	−0.26	[−0.39, −0.13]	**< 0.001**
	Strongly disagree	3.09 (0.51)	−0.40	[−0.62, −0.19]	**< 0.001**
Attitude 3: Engaging with treatment is a waste of time and won't help.	Disagree	2.75 (0.50)	—	—	—
	Agree	2.99 (0.45)	−0.24	[−0.35, −0.12]	**< 0.001**
	Strongly agree	3.14 (0.63)	−0.39	[−0.60, −0.18]	**< 0.001**
	Strongly disagree	2.50 (0.56)	0.25	[0.18, 0.32]	**< 0.001**
Attitude 4: Mental health problems can be effectively treated.	Agree	2.67 (0.52)	—	—	—
	Strongly agree	2.56 (0.59)	0.11	[0.32, 0.19]	**0.003**
	Disagree	2.89 (0.51)	−0.23	[−0.33, −0.13]	**< 0.001**
	Strongly disagree	3.17 (0.58)	−0.50	[−0.71, −0.29]	**< 0.001**

The belief that mental health problems cannot be effectively treated was associated with significantly higher levels of self‐stigma (Attitude 4 response ‘disagree’, *t*(694) = −4.547, *p* < 0.001 and ‘strongly disagree’, *t*(587) = −4.701, *p* < 0.001, reference category ‘agree’), and the belief that engaging with treatment will not help was also associated with significantly higher levels of self‐stigma (Attitude 3 response ‘agree’, *t*(564) = −4.012, *p* < 0.001 and ‘strongly agree’, *t*(506) = −3.717, *p* < 0.001, reference category ‘disagree’).

Differences were not seen in attitudes to treatment among people who did and did not have a personality disorder diagnosis, except in relation to the proportion of people with personality disorder who endorsed the item that their mental health condition can be effectively treated, which was higher among people with a diagnosis of personality disorder compared with those without the diagnosis (*χ* ^2^ (3, *N* = 1009) = 11.76, *p* = 0.008).

## 4 Discussion

The results of this study provide new evidence about levels of self‐stigma among people who have been diagnosed with personality disorder compared with people diagnosed with other common nonpsychotic mental health conditions in the United Kingdom. They also highlight factors associated with self‐stigma and the relationship between self‐stigma and beliefs about treatment. We found that levels of self‐stigma were higher among people who had been given a diagnosis of personality disorder, compared with people diagnosed with anxiety and depressive disorders. However, having a diagnosis of personality disorder was not associated with levels of self‐stigma once the effects of personality dysfunction, depressive symptoms and other confounding variables were considered. The strongest predictor for levels of self‐stigma was the severity of depressive symptoms. Occupational status, level of education and self‐reported exposure to invalidating childhood environments were also associated with the level of self‐stigma that people reported. People with higher levels of self‐stigma also reported having less favourable beliefs about the value of treatment for their mental health condition. Attitudes to treatment were similar whether people had been given a diagnosis of personality disorder or not.

### 4.1 Factors Associated With Self‐Stigma and Their Impact

The main finding from this study is that, in contrast to our initial hypothesis, being given a diagnosis of personality disorder was not associated with the overall level of self‐stigma once other factors such as depressive symptoms and level of personality dysfunction were accounted for. Although there is limited evidence from previous studies, these studies have reported higher levels of self‐stigma among people with a personality disorder diagnosis compared with people with other psychiatric diagnoses such as ADHD, bipolar disorder and anxiety (Grambal et al. 2016; Quenneville et al. 2020). However, these studies did not explore the relationship between personality disorder diagnosis and level of personality dysfunction on levels of self‐stigma. These findings suggest that rather than the process and consequences of being diagnosed with personality disorder having an impact on levels of self‐stigma, it is aspects of personality dysfunction such as disturbed sense of self and impaired interpersonal functioning that influence the level of self‐stigma that people experience.

Although the relationship between self‐stigma and severity of depression and levels of educational achievement are well established (Holubova et al. 2016; Yen et al. 2005), this is the first study to our knowledge to examine the impact of exposure to invalidating childhood experiences on levels of self‐stigma in people with personality disorder. Our finding that self‐reported exposure to invalidating childhood environments is linked to levels of self‐stigma points to the importance of early life experiences may have in the way that negative beliefs about the self may evolve. Although research into the association between invalidating childhood experiences and self‐stigma has not been extensively conducted, it can be hypothesised that invalidating childhood experiences can have a negative impact on a person's sense of self, contributing to higher levels of self‐stigma. Invalidating childhood experiences may lead to future negative self‐perception and self‐invalidation (Musser et al. 2018) and the internalisation of negative beliefs associated with oneself. It may also cause individuals who have experienced these environments to be more self‐critical (Naismith, Zarate Guerrero, and Feigenbaum 2019), which could lead to an increased likelihood of them internalising stigmatising views associated with their mental health condition and subsequently believing them to be true. The results in this study highlight the potentially lasting impact of childhood experiences on individuals' self‐perception and internalised stigma.

The strongest predictor for levels of self‐stigma was the severity of depressive symptoms. This is in line with previous research, with Gilkes, Perich, and Meade (2019) finding that depression was significantly associated with self‐stigma. This can be interpreted from a bidirectional perspective. If an individual is depressed, they may be more likely to experience feelings of worthlessness, which makes it more likely that they would internalise stigmatising attitudes and beliefs.

Compared with being unemployed, being in full‐time employment was significantly associated with lower self‐stigma, as was being in part‐time employment, being a student or retired. Holubova et al. (2016) also found that participants who were unemployed tend to experience a higher level of self‐stigma than those in employment, suggesting that employment may be a protective factor against self‐stigma. Individuals who are in employment may feel a greater sense of purpose (Perkins et al. 2009), like they are contributing to society and have control over their lives despite their mental illness and any associated stereotypes, increasing their self‐esteem and sense of self‐worth and reducing self‐stigma. These individuals are also more likely to form social connections and therefore less likely to become withdrawn and isolated.

Higher level of education appears to be a protective factor against self‐stigma. Primary education was found to be associated with higher levels of self‐stigma, whereas university‐level education was associated with lower levels of self‐stigma. This is in line with Yen et al. (2005) who found that a lower level of education is associated with higher self‐stigma and Holubova et al. (2016) who found that participants with university‐level education had lower levels of self‐stigma than those who only completed up to secondary level education. Level of education may act as a protective factor against self‐stigma as pursuing further education may lead to individuals having a greater general understanding of mental health, thus leading to increased mental health literacy, making them more able to identify and challenge negative stereotypes surrounding mental health. This may lead to a greater sense of empowerment in individuals and allow them to proactively challenge any self‐stigmatising thoughts.

The results of this study provide additional evidence of an association between self‐stigma and attitudes towards treatment (Gaudiano and Miller 2013; Latalova, Kamaradova, and Prasko 2014). In contrast, we found little difference in attitudes towards treatment between people diagnosed with personality disorder and people diagnosed with other common mental health conditions.

### 4.2 Strengths and Limitations

This is the largest study to have been conducted which has examined levels of self‐stigma among people with personality disorder. By recruiting members of the general public, we have been able to avoid the potential of survivorship bias in studies of people in contact with mental health services, where those with the highest levels of self‐stigma may be less likely to maintain contact with services.

Further strengths of conducting an online survey are that it enabled us to have wide geographic reach from across the country and to collect data from a large enough sample to be able to examine the study hypothesis in a timely manner. By using the same advert and online platforms to recruit both those with personality disorder and those without, we were able to ensure that the data we collected from people with personality disorder were compared with those with other common mental health conditions.

The study also has some limitations. Firstly, by solely using social media platforms for recruitment, we excluded those people who do not use social media. However, it has been estimated that over 80% of the UK population are active social media users (Kemp 2024), and social media is increasingly being used to recruit populations who may be otherwise hard to reach, as well as more easily allowing for geographical diversity (Darko, Kleib, and Olson 2022).

The study sample was mainly female, and although women also make up the majority of people with personality disorder who are in contact with mental health services (Dyer 2016; Paris 2004), we do not know if these results would generalise to men with personality disorder.

As with all cross‐sectional studies, we do not know whether the associations we found are causal. For instance, it is possible that, rather than exposure to invalidating childhood environments leading to greater self‐stigma, it may be that people with higher levels of self‐stigma are more likely to recall exposure to experiences of invalidating during their childhood.

Our use of short versions of assessment tools meant that we do not have data from gold standard measures of depression, personality disorder and other exposure variables. However, the measures that we used do provide reliable and valid assessments of the factors we examined, and we judged that the use of lengthier versions of measures would have impeded our ability to collect data from the large sample of participants that we needed to recruit to the study.

Finally, our use of the SAPAS to measure the severity of personality difficulty is also a limitation. The SAPAS does not fully map onto the ICD‐11 level of severity construct. The SAPAS primarily focuses on interpersonal functioning, potentially overlooking other crucial aspects of personality functioning. This narrow focus may have influenced our results, as measures of personality functioning that include self‐functioning domains could potentially demonstrate even stronger predictive power for self‐stigma.

Additionally, our reliance on the SAPAS may have limited our ability to capture the full spectrum of personality dysfunction. Future studies should consider employing more comprehensive measures of personality functioning that encompass both interpersonal and self‐functioning domains. This approach would provide a more holistic assessment and potentially yield more robust insights into the relationship between personality dysfunction and self‐stigma.

### 4.3 Implications for Future Research and Practice

Although the results of this study highlight the significance of self‐stigma among people with personality disorder, further research is needed to better understand the causes and impact of self‐stigma among people with personality disorder. Longitudinal studies have the potential to track changes in levels of self‐stigma among people with personality disorder from when they first make contact with healthcare services. Such studies could quantify the impact that contact with mental health services has on self‐stigma and establish what impact high levels of self‐stigma have on whether people engage with and benefit from psychological and psychosocial interventions. Such research would benefit from recruiting both male and female participants with a wide range of educational backgrounds and comorbid conditions, as well as examining the impact that early life experiences may have on levels of self‐stigma before people have experience of using healthcare services. Qualitative research will also be important in understanding the experiences of people with personality disorder and developing ways to mitigate against the impact that self‐stigma has on providing effective treatment and support.

Meanwhile, clinicians working with people with personality disorder should be aware of high levels of self‐stigma among people with personality dysfunction. The impact of self‐stigma on whether people are able and willing to accept offers of treatment and support should be explored. Furthermore, clinicians should work towards providing more individualised treatment pathways that prioritise an individual's subjective personal experiences and their related functional impairments, as doing so may help individuals develop protective mechanisms against self‐stigmatising beliefs and attitudes. For example, taking a strengths‐based approach by which individuals feel more empowered and are able to develop a stronger sense of self‐concept may lead to better outcomes and reduced self‐stigma.

Finally, public mental health literacy associated with personality disorder is poor (Furnham, Lee, and Kolzeev 2015; Furnham and Winceslaus 2012). There is evidence that public campaigns aimed at improving mental health literacy have helped reduce the stigma associated with depression and other mental health conditions. In the United Kingdom, the Time to Change campaign was found to be associated with greater levels of awareness, support and tolerance of mental disorders such as depression (Sampogna et al. 2017). These results suggest that future antistigma campaigns should include a focus on people with poor mental health associated with their personality. Sirey et al. (2001) also suggest placing focus on providing information that helps individuals identify stigma and develop coping mechanisms to counteract it, thus potentially increasing the likelihood of people seeking support for their mental health, experiencing less self‐stigma and more positive attitudes towards treatment.

## Conflicts of Interest

The authors declare no conflicts of interest.

## Data Availability

The data that support the findings of this study are available from the corresponding author upon reasonable request.
